# The effect of whey protein ingredient substitution on proteolysis stability of model high-protein beverage: A pilot study

**DOI:** 10.3168/jdsc.2026-1092

**Published:** 2026-08-06

**Authors:** Tyler R. Jarrard, Qihui Wu, Hari Meletharayil, Haotian Zheng

**Affiliations:** 1 Food Rheology Laboratory, Department of Food, Bioprocessing and Nutrition Sciences, North Carolina State University, Raleigh, NC 27695; 2 Southeast Dairy Foods Research Center, North Carolina State University, Raleigh, NC 27695; 3 Dairy Management Inc., Rosemont, IL 60018

## Abstract

The shelf life of high-protein beverages made from concentrated milk protein ingredients can be limited by the activity of the indigenous protease, plasmin. Plasmin (PL) is known to survive pasteurization and withstand UHT treatment temperatures. Previous works have shown that β-LG has a potential to inhibit PL activity. The effect of partial protein substitution (∼7%–20%) using various whey protein ingredients in model high milk protein dispersion on potential PL inactivation and proteolysis inhibition was investigated in the present work. Model high-protein dispersions (7.5% total protein) were prepared using milk protein concentrate with 85% protein content by mass (MPC85), 2 types of whey protein isolate (WPI), and whey protein hydrolysate (WPH) ingredients. The whey protein ingredients were used to substitute proteins in the control MPC85 dispersion. The PL activity and age proteolysis effect were characterized before and after an oil-bath heat treatment (95°C for 100 s) on individual protein dispersions. Although partial WPI substitution resulted in a lower apparent PL activity compared with the MPC control, the difference became insignificant after heat treatment. Moreover, the effect toward preventing casein proteolysis was insignificant up to 20% protein substitution using a standard WPI and a β-LG–enriched WPI, respectively. Interestingly, WPH substitution resulted in increased apparent PL activity in protein dispersions after heat treatment compared with the MPC control. No significant difference was observed between the control and any of the substituted samples after heat treatment for age proteolysis; samples showed ∼70% to 90% retention of intact α_S1_-CN with no significance after 3 d of accelerated storage (37°C). The obtained results suggest that the applied heat treatment on the milk protein dispersions was more effective than whey protein substitutions on preserving plasmin system induced proteolysis.

## Graphical Abstract

**Figure:**
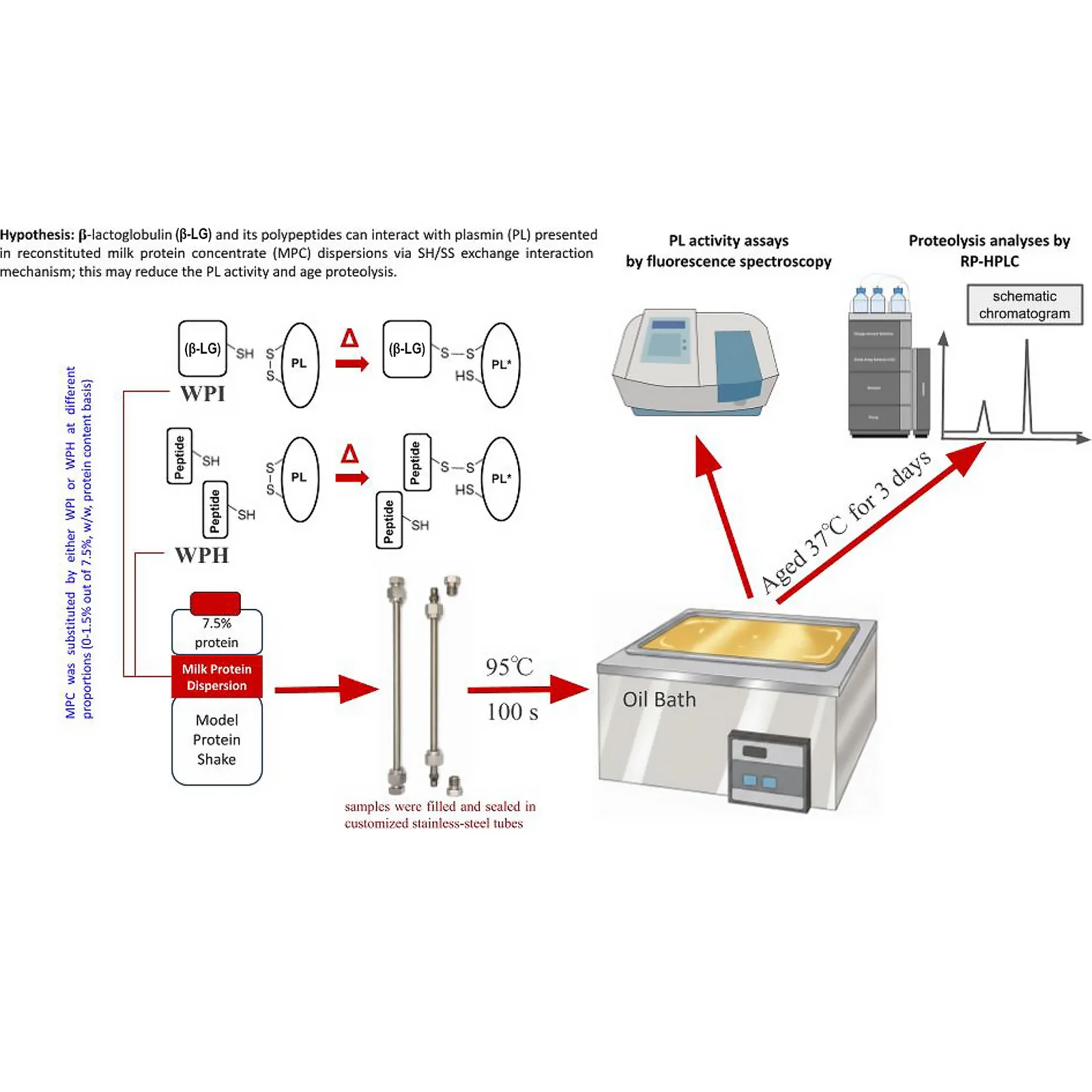

The plasmin protease system is among the most important endogenous enzymes present in milk products and is problematic for casein stability, upon which it has proteolytic activity. The appearance of the γ-CN (proteolysis products of β-CN) in fresh milk is evidence that plasmin (**PL**) activity is already underway before milking (Bastian and Brown, 1996). After typical pasteurization conditions (72°C for 15–30 s) PL activity not only remains but increases due to partial inactivation of its inhibitors and enhanced susceptibility of inactive plasminogen (**PG**) to the likewise heat-stable plasminogen activators (**PA**; Dulley, 1972; Kelly and Foley, 1997; Prado et al., 2006). Plasmin activity in dairy ingredients poses limitations to their utilization. In shelf-stable ready-to-drink (**RTD**) beverages, PL-proteolysis limits their shelf life, causing age-induced defects such as physical separation and the onset of bitter flavors (Rauh et al., 2014b).

Previous studies have demonstrated the influence of filtration-fractionation of PL activity and proteolysis in dairy products and ingredients (Aaltonen and Ollikainen, 2011; Gazi et al., 2014; Puri et al., 2020; Jarrard et al., 2026). In general, PL and PG are associated with casein micelles, and PL activity is expected to be retained in filtered products. Thus, PL activities are typically high in microfiltered micellar casein concentrates (**MCC**), micellar casein isolates (**MCI**), UF milk protein concentrates (**MPC**), but lower in sweet whey protein ingredients (Hayes and Nielsen, 2000; Crudden et al., 2005). Gazi et al. (2014) reported that reconstituted protein dispersions made from MCI and MPC, respectively could experience significant casein hydrolysis (e.g., >80% under an extreme storage condition for an accelerated shelf-life study). This finding suggests that high-protein beverages made from MPC may have a high potential for proteolysis; therefore, proper thermal inactivation of PL and other key PL system components are critical to the proteolytic stability of the beverage products. Due to the complexity of interactions among milk components, especially the PL system and milk proteins, effective PL inactivation approaches for high-protein formulas are not yet available. Actual or apparent inhibition of PL activities by constituent milk proteins has been investigated in different model milk product systems (Bastian et al., 1993; Politis et al., 1993; Puri et al., 2020; Jarrard et al., 2026); nevertheless, reconstituted MPC systems mimicking high-protein beverages are still understudied with regard to PL inactivation and improvement of proteolytic stability after heat treatment. By comparing PL inactivation parameters (D-value) reported from prior works, it seemed that PL in MPC dispersion systems with fortified protein content were relatively more heat resistant compared with milk dispersions with regular protein contents (Deeth, 2021; Jarrard et al., 2027). Consequently, such high-protein beverages made with MPC may require higher intensity heat treatment (e.g., higher temperature and longer time) to inactivate PL activity, thus producing the potential for thermal abuse and thermally induced product instability. Particularly, this might be a processing issue for high-protein beverages made from highly concentrated MPC ingredients (e.g., it was reported that high-protein dispersions [8.5% protein content] made from MPC85 and MPC90 [MPC powders with 85% and 90% protein content by mass, respectively] had poor heat stability at pH 6.8 compared with those made from lower protein content MPC, such as MPC70 and MPC80; Crowley et al., 2015). Therefore, an optimum and a dedicated heat treatment is needed for high-protein dispersion made from MPC85 for effectively inactivating PL activity without causing a physicochemical instability. Moreover, it has been reported that the addition of β-LG, proteins with free thiol (-SH) group, was beneficial for accelerated PL inactivation (Alichanidis et al., 1986; Saint-Denis et al., 2001a). Interestingly, in conditions where β-LG loses its capacity to inactivate PL, such as by chemical modification or at low pH, the stability of PL is once again enhanced (Alichanidis et al., 1986; Kelly and Foley, 1997; Crudden et al., 2005). As it stands, however, it is unclear whether enrichment of β-LG or whey proteins will have a substantial effect on PL inactivation in a fortified protein dispersion system made from highly concentrated MPC (e.g., MPC85).

The potent PL-inactivating behavior of β-LG is attributed to the nucleophilicity of its free thiol, explaining both the association between inactivation potential with unfolding kinetics and pH (Alichanidis et al., 1986; Metwalli et al., 1998; Saint-Denis et al., 2001a; Crudden et al., 2005). Sulfydryl-containing compounds, such as cysteine and glutathione, are known to rapidly accelerate PL and PG inactivation at low concentrations (2.0–1.0 m*M*) but without the unfolding-dependent kinetics associated with β-LG (Metwalli et al., 1998; Lu et al., 2009; Ryan et al., 2012). In Ryan et al. (2012), it was demonstrated that added thiolates resulted in the catalytic polymerization of PA, suggesting that thiol-disulfide exchange reactions prohibit activation of PG and activity of PL. However, as these small thiolate compounds are prone to generate sulfurous flavors during heat treatment, their use is precluded in most commercial applications (Arnold, 1969). Although nonsulfydryl reducing agents could also be used, such as sodium borohydride, this required far greater concentration to achieve a similar effect (Ryan et al., 2012). Consequently, there is a need to discover food ingredients that have sufficient thiolate content to inactivate the PL system in systems relevant to milk products without the development of small flavor molecules. It is reasonable to hypothesize that after hydrolysis of whey proteins (majorly β-LG), free thiol groups become exposed and more accessible (Caessens et al., 1999). Therefore, whey protein hydrolysate (**WPH**) is an ingredient worth investigating for its effect on thermal inactivation of PL in high-protein dispersions. Therefore, this work aimed to investigate the effect of whey protein ingredient substitution on the thermal inactivation of key PL system components (PL and PA) in a model high-protein dispersion mimicking a RTD beverage base formula consisting of MPC85 as the primary protein ingredient. The heat-treated sample dispersions were placed in an extreme storage condition for stimulating accelerated proteolysis, thus, the efficacy of the conducted thermal inactivation of the PL system may be characterized.

To prepare samples, freshly pasteurized skim milk was obtained from the local dairy processing pilot plant at North Carolina State University (Raleigh, NC). Bovine PG was purchased from Sigma-Aldrich (Saint Louis, MO). Fluorescent substrate N-succinyl-ala-phe-lys 7-amido-4-methylcoumarin acetate salt and its hydrolysate 7-amino-4-methylcoumarin were obtained from ThermoFisher (Waltham, MA). Materials for the clarifying reagent were 2-butanone (Sigma-Aldrich), Triton X–100 (Sigma-Aldrich), SDS (ThermoFisher Scientific), and NaOH (ThermoFisher Scientific). All other materials were sourced from Sigma-Aldrich or ThermoFisher as HPLC or analytical grade.

The MPC85 powder (83.8% protein) used in this study was produced in house as described previously (Jarrard et al., 2027). Standard whey protein isolate (**WPI** group, 89.5% protein, WPI 9000), β-LG–enriched whey protein isolate (88.6% protein, WPI 9400, namely the β-LG group), and WPH (76.9% protein, WPH 8390) are commercial ingredients sourced from Hilmar Cheese & Ingredients (Hilmar, CA). The β*-*LG ingredient had ∼19% β-LG enrichment compared with the standard WPI (WPI 9000) and “WPH” had ∼97% and ∼99% reductions in β-LG and α-LA compared with the standard WPI (WPI 9000) as analyzed by gel electrophoresis (SDS-PAGE with reducing condition). These ingredients were reconstituted as protein dispersions (7.5% protein, wt/wt) according to Table 1 in deionized water. Two replicates were produced for each model beverage. The pH for each beverage was between 6.8 and 6.9.

**Table 1:** Physical properties of MPC85 protein dispersions^1^

Sample group	Protein content^2^ (%, wt/wt)		Hydrodynamic radius^3^ (nm)		ζ-Potential^3^ (mV)	
	MPC	Substitute	Fresh	Heated	Fresh	Heated
MPC	7.5		49.6 ± 2.5^a^	44.1 ± 2.1^b^	−41.2 ± 2.4^a^	−34.7 ± 4.8^a^
WPI 1	7.0	0.5	49.6 ± 1.2^a^	45.1 ± 2.1^a^	−42.1 ± 0.0^a^	−41.8 ± 0.6^a^
WPI 2	6.5	1.0	53.6 ± 2.7^a^	58.7 ± 5.6^a^	−35.9 ± 3.6^a^	−39.0 ± 7.8^a^
WPI 3	6.0	1.5	57.0 ± 6.0^a^	57.3 ± 17.0^a^	−41.3 ± 7.7^a^	−41.2 ± 8.4^a^
β-LG 1	7.0	0.5	50.9 ± 4.8^a^	48.2 ± 3.6^a^	−42.8 ± 0.1^a^	−43.4 ± 0.6^a^
β-LG 2	6.5	1.0	51.5 ± 0.9^a^	37.7 ± 2.1^b^	−39.3 ± 4.7^a^	−40.2 ± 5.0^a^
β-LG 3	6.0	1.5	53.1 ± 0.8^a^	41.9 ± 1.6^b^	−38.6 ± 1.2^a^	−35.9 ± 3.4^a^
WPH	7.25	0.25	58.1 ± 0.6^a^	56.8 ± 8.8^a^	−39.4 ± 1.4^a^	−36.4 ± 0.4^a^

a,b Means in a row that do not share the same superscript letters are significantly different (*P* < 0.05) according to paired sample *t*-test.

1 Data represent the mean ± SD (n = 2).

2 Protein contents contributed by different protein ingredients between MPC (control ingredient) and 3 different substitute ingredients: standard WPI (WPI), β-LG (β-LG–enriched WPI), and whey protein hydrolysate (WPH).

3 No significant differences were noted (*P* > 0.05) among samples within the same column according to one-way ANOVA analysis.

Samples were heated in a hot oil bath at 95°C for 100 s using the equipment setup described in Jarrard et al. (2027). A portion (2.0 mL) of each liquid beverage was transferred to individual 18-cm 316-grade stainless steel tubes with an outer diameter of 6.35 mm and an internal diameter of 4.5 mm, subsequently, the sample tubes were sealed hermetically. The oil bath was superheated by 3°C above the desired heating temperature to accommodate the instantaneous cooling associated with adding the sample rack and metal tubes, with the final oil temperature being recorded as 95.5°C. After heating for the designated time (100 s), the samples were removed at once and cooled in an ice bath. Following cooling, the liquid samples were removed and frozen at –80°C until further analysis. Fouling and sedimentation were not observed in any samples. The particle size of each sample before and after heat treatment is shown in Table 1. The particle size in the control sample is consistent with known ranges for MPC data (Sønderby et al., 2025).

The PL and PA activities were determined using the methodology prescribed by Saint-Denis (2001a; b) with reduced reaction volumes as described previously (Jarrard et al., 2026). Briefly, the PL and PA activities were indicated by the amount of 7-amino-4-methylcoumarin (**AMC**) being generated in the assays as explained in the referred works. One AMC unit of PL activity (**PL-U**) is defined as the amount of enzyme that produced one nanomole of AMC per milliliter of reaction mixture per minute at 37°C (1 PL-U = 1 nmol AMC·min^−1·^mL^−1^) with baseline AMC hydrolysis subtracted. One AMC unit of PA activity (**PA-U**) is defined as the amount of enzyme that increased the rate of AMC production by one nanomole per milliliter of reaction mixture per minute at 37°C (1 PA-U = 1 nmol AMC·min^−2^·mL^−1^) with baseline PL activity removed. Because the WPI, β-LG–enriched WPI, and WPH solutions themselves—from which the samples in Table 1 were made—were not observed to have significant levels of PL or PA activity (data not shown), PL and PA activities were recorded on a basis of protein content from MPC. All enzymatic assays were performed in duplicate.

To quantify actual protein integrity during storage, the protein profiles of all samples after heat treatment and after 3 d of accelerated aging (37°C) was observed using reverse-phase HPLC using the method described previously (Hinz et al., 2012; Gazi et al., 2014; Jarrard et al., 2026) with peaks identified as described by Visser et al. (1991). Percent intact protein was defined as the ratio of protein peak area before and after storage, with α_S1_-CN being used as an indicator for hydrolysis overall (Jarrard et al., 2026). ζ-potential and particle size distribution were determined using dynamic light scattering (Mobius, Wyatt Technology; 0.1% sample vol/vol in ultrapure water).

One-way ANOVA was used to calculate differences of apparent PL and PA activity between different milk protein dispersions in unheated control samples, as well as comparing those same samples after heat treatment, with letter differences determined with post hoc pairwise comparison (Tukey's test) using OriginPro software (Northampton). One-way ANOVA was also performed to determine differences of α_S1_-CN retention after storage for fresh and heated samples. One-way ANOVA and “paired sample t-test” was performed to determine any differences in physical characteristics (particle size and ζ-potential) for each sample, before and after heating. Letter differences were determined with post hoc pairwise comparison (Tukey's test). The significance level was set as 0.05.

Substituting MPC by 2 types of WPI regardless of the proportions resulted in reduced apparent PL activities relative to the MPC control in unheated samples (Figure 1A, *P* < 0.05). However, enriching WPI or β-LG in the reconstituted MPC dispersion did not affect the apparent PA activity (Figure 1B). These observations are in partial agreement with a previous study, in which the authors showed that in a protein system without heat treatment, native β-LG showed no effect on PA activity and would reduce PL activity depending on the assay substrate used (Rippel et al., 2004). It is critical to note that the “apparent” inhibition effects induced by WPI substitutions might be caused by whey protein interference to the assay performed rather than a true “inhibition” effect. In detail, the interference might be derived from an altered “ligand-binding” effect in the enzymatic assay. It is reasonable to speculate that the enriched whey proteins interacted with the assay substrate, and consequently, the PL-substrate interactions were reduced and the “apparent” PL activity as measured by the assay was reduced. Such whey protein–induced assay interference was reported in literature, in which both native β-LG and α-LA showed significant “ligand-binding” effects in association with an assay substrate (Rippel et al., 2004). However, regarding PA activity in fresh samples, WPI substitution showed no effect as compared with the MPC control regardless of the substitution level (Figure 1B). This suggests that the apparent “inhibition” as induced by whey protein-assay substrate interference effect had been offset by an enhanced PA activity as stimulated by the enriched whey proteins. Rippel et al. (2004) found that native α-LA, denatured α-LA, and β-LG were able to enhance PA activity.

**Figure 1:**
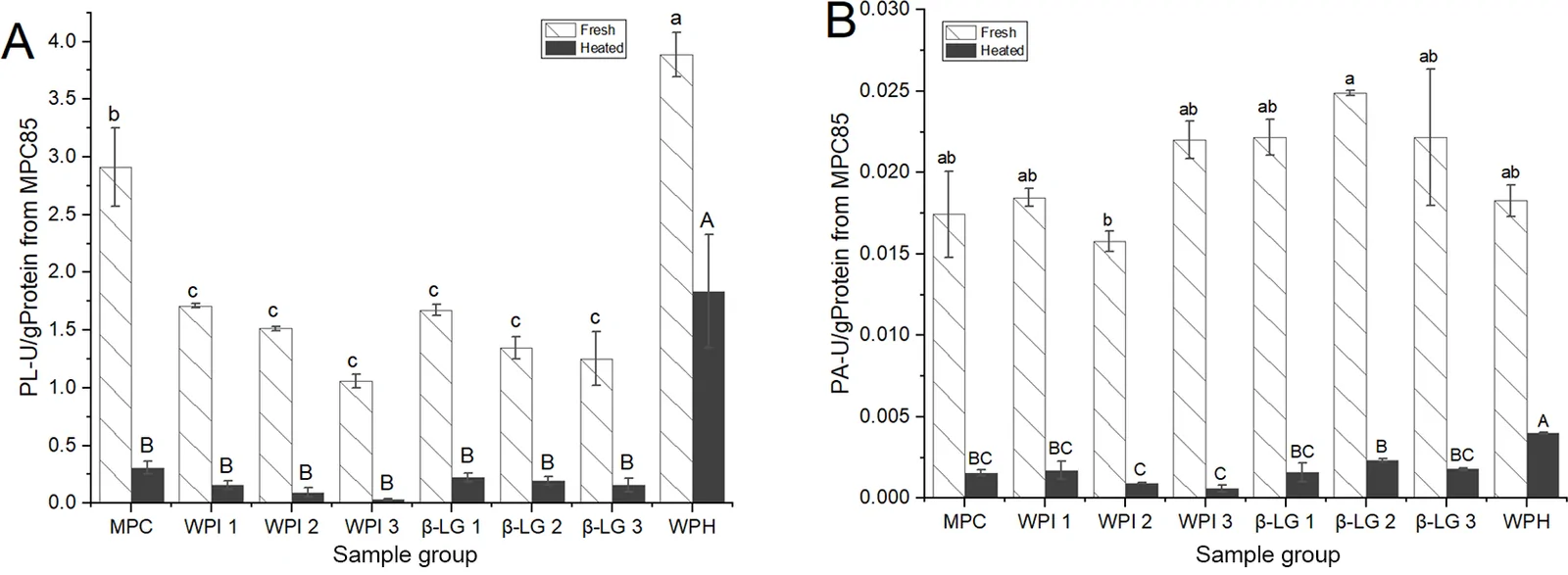
(A) Plasmin and (B) PA activities for MPC85 model dispersions (7.5% total protein) with added (wt/wt) whey protein isolate (WPI), β-LG–enriched WPI (β-LG), and whey protein hydrolysate (WPH); before and after heat treatment. Data represent the mean ± SD (n = 2). The PL activity reductions before heating are likely inhibitory, with losses primarily due to inactivation afterward. Whey protein hydrolysate appears to either enhance or protect PL activity. The PA inactivation does not seem greatly affected by additional whey protein. Letter designations indicate statistical comparison information according to one-way ANOVA using Tukey's test. Means that do not share the same letter are significantly different (*P* < 0.05).

For heat-treated samples, WPI substitutions showed no significant effect on PL activity compared with the MPC control (Figure 1A). Prior studies suggested the role of β-LG in PL inhibition and inactivation in heat-treated systems (Bastian et al., 1993; Politis et al., 1993). Therefore, it was expected that an increased relative proportion of β-LG would result in an enhanced PL inhibition induced by thermal inactivation. However, this phenomenon was not observed in the results shown in Figure 1A regardless of the WPI substitution level. Heat treatment induced massive reductions in PL and PA activities for all the tested samples (Figure 1); this is to a certain extent aligned with our previous finding, in which 95°C/100 s would be able to induce inactivation of PL and PA (Jarrard et al., 2027), whereas Figure 1 shows the WPI substitutions did not substantially affect the apparent activities of PL and PA after a relatively moderate heat treatment. This finding is in partial disagreement with the current literature knowledge on milk systems; it had been suggested that native and denatured ꞵ-LG may inhibit PL activity in both reconstituted casein and bovine milk systems after a heat treatment (Bastian et al., 1993; Saint-Denis et al., 2001a). The different observations in the present work could likely be attributed to the differences in the protein dispersion samples compared with the ones used in previous works. The present samples were made from protein concentrate and protein isolates (MPC and WPI groups) having reduced the ionic strength, whereas the samples used in the cited works had an ionic strength comparable to the original skim milk. Therefore, the electrostatic “screening effect” was significantly reduced in current samples compared with the ones used in the cited works (Saint-Denis et al., 2001a). This is evidenced by the ζ-potential results shown in Table 1, where the surface charges of these samples are higher than a typical skim milk, which would have a ζ-potential <−20 mV (Marine Philippe et al., 2003; Markoska et al., 2019). According to the Derjaguin–Landau–Verwey–Overbeek (DLVO) theory, a stronger protein-protein repulsion can be expected from a lower screening effect or a higher surface charge. Moreover, it may be speculated that heat-induced denaturation of whey proteins may have altered the whey protein interference effect on the assay substrate.

In addition, since the concentration of whey proteins in the MPC control was relatively high already, due to the high-protein content in model dispersions, it is likely that a near-saturation condition had been achieved in the control sample with fortified protein content, in which additional whey protein content would not be able to induce additional inhibition effects on PL. Prior research on whey protein inhibition of the PL system in buffers observed that at β-LG concentrations present in milk (4–6 mg/mL), PL activities were already inhibited by 78%, compared with 53.8% at 1 mg/mL and 18.2% at 0.2 mg/mL (Politis et al., 1993). This is similar to the effect on the removal of whey proteins, in which it was previously observed that PL activities rise during microfiltration, as β-LG and α-LA are removed (Aaltonen and Ollikainen, 2011). The PA activities varied less than those for PL (Figure 1B). The WPI substitutions resulted in neglectable effect on PA activity against the MPC control regardless of substitution level and heat treatment. The cause of the identical PA thermal inactivation level between MPC and WPI substituted sample groups is analogous to the PL inactivation mechanisms as discussed above; plus, the heat-denatured whey protein might have lost its functionality in stimulating PA as suggested in the literature (Rippel et al., 2004).

Interestingly, WPH substitution led to elevated “apparent” PL activities compared with the control, MPC dispersions, regardless of heat treatment (*P* < 0.05). As no PL or PA activity was observed in the WPH ingredient control itself (data not shown) the elevated PL activity can be likely attributed to the assay interference effect. It has been widely accepted that both whey proteins and caseins can interfere with PL assay causing underestimated activity (Bastian et al., 1993; Saint-Denis et al., 2001b; Rippel et al., 2004; Rauh et al., 2014a). However, the interference effect of the polypeptides in WPH is still not clear. It may be speculated that peptides in the WPH group had reduced binding capacity with assay substrate or reduced interference with the assay in general. Therefore, more assay substrates were available to the PL in fresh and heated protein dispersions resulting in elevated PL activities (Figure 1A). The WPH substitution did not show an effect on PA activity in fresh protein dispersion, even though WPH probably caused less assay interference. However, the WPH enriched sample with depleted intact whey proteins compared with the MPC control dispersion, would have reduced the stimulation effect on PA activity as discussed above. Therefore, the suppressed PA stimulation and reduced assay interference offset each other. Whereas for the heat-treated samples, the PA activity was higher in the sample substituted with WPH (*P* < 0.05, Figure 1B). The true cause of this phenomenon is unknown, but it is likely related to the characteristic functionalities of denatured WPH as a result of the heat treatment.

A short, accelerated aging period was used here (37°C, 3 d) to examine the proteolysis of both heated and unheated (fresh) samples, as shown in Figure 2. The fresh samples except the one with WPH substitution yielded a broad range of residual α_S1_-CN levels ranging from ∼22% to ∼46%; however no significance was observed (Figure 2, *P* > 0.05). The low level of protein retention (∼22%) in MPC control after storage was comparable to our prior results (∼25%) in newly manufactured MPC dispersion systems (Jarrard et al., 2026). It was observed that the highest WPI substitution in the fresh sample resulted in a higher α_S1_-CN retention compared with the WPH-containing dispersion (*P* < 0.05), with ∼46% and ∼12% residual protein after the accelerated storage. This observation of proteolysis does not seem to be directly associated with the results of PL and PA activities shown in Figure 1. Such observation served indirect evidence that the results obtained from the enzymatic assays had been affected by an interference effect. No significance was observed among all heated samples for the residual α_S1_-CN (Figure 2). Consequently, it can be concluded that the enrichment of whey protein and WPH contents in MPC dominated high-protein beverage systems cannot distinctively inhibit casein proteolysis. The applied heat treatment effectively improved casein retention as indicated by the residual α_S1_-CN ranging from ∼70% to ∼90%. A considerable SH/SS exchange was reported for a PL hydrolysis of ꞵ-LG and the peptide fragments were linked via disulfide bridging (Caessens et al., 1999). Moreover, it was reported that depending on the protease used, the SH group content could be reduced in a WPH (Mohan et al., 2015). These literature results suggest a possibility that the amounts of accessible and total thiol groups in WPH were not necessarily enriched or had been oxidized during ingredient production. Therefore, the substituted WPH peptides may not necessarily be more reactive with PL system components via SH/SS shuffling as compared with the WPI ingredients. Consequently, it can be concluded that the enrichment of whey protein and WPH contents in MPC dominated high-protein beverage systems cannot result in a distinctive effect on inhibiting casein proteolysis. Total and (surface) accessible SH groups in WPH ingredients need to be characterized in future works for investigating their inhibitory effects on PL.

**Figure 2:**
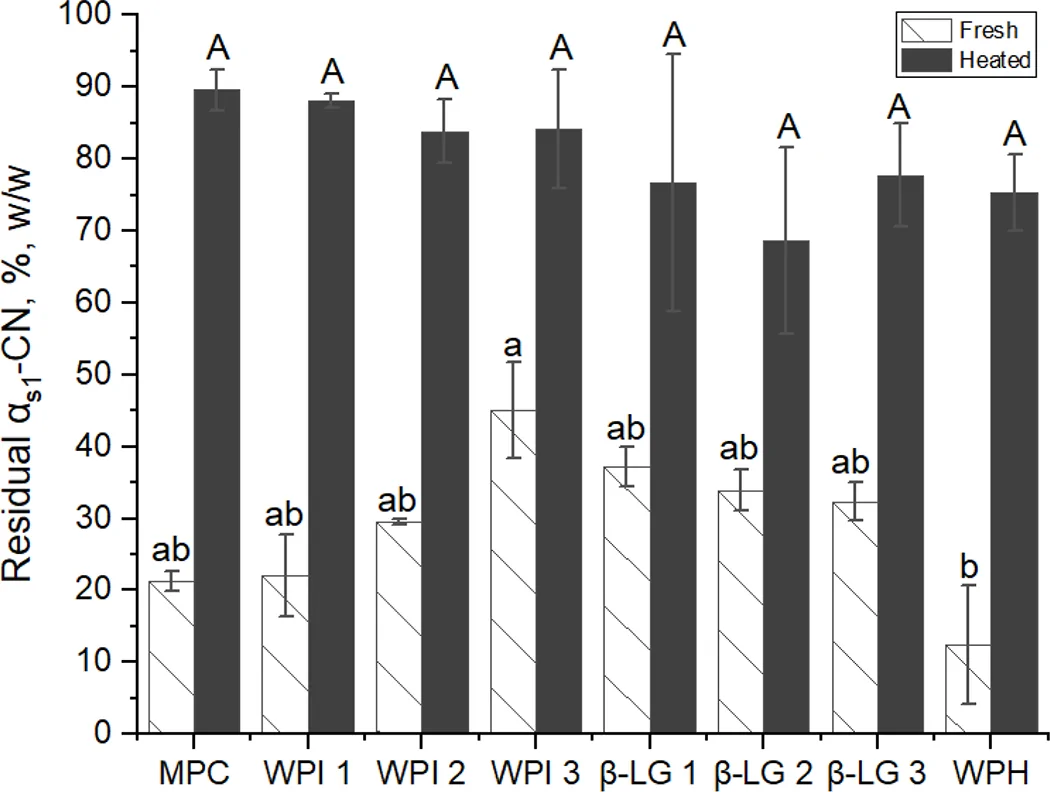
Intact α_S1_-CN relative peak areas in fresh MPC85 protein dispersions heated at 95°C for 100 s after 3 d of accelerated storage at 37°C. Relative peak areas shown are compared with the same sample before storage. Letter designations on top of each bar indicate statistical comparison information according to one-way ANOVA using Tukey's post hoc test. The result bars and error bars are plotted based on means and SD, respectively; means that do not share the same letter are significantly different from the MPC control (*P* < 0.05), with lowercase letters for fresh sample peak areas after storage and uppercase letters for heated sample peak areas after storage (*P* < 0.05).

The present work provided practical results, showing that the implemented heat treatment significantly reduced PL activity and proteolysis in a model high-protein beverage dominated by milk proteins. This finding justifies an opportunity of inactivating PL activity and preventing age-induced proteolysis via an optimized thermal inactivation approach. Thus, thermal inactivation is worthy of more in-depth studies for optimized effects on prevention of proteolysis in RTD high-protein beverages. Particularly, future works are recommended on optimizing preheating conditions on high-protein model systems such as using relatively lower temperature (e.g., <100°C) with a proper holding time; this research direction is suggested to be built based on the findings from the present work and few previous relevant works (e.g., Newstead et al., 2006; Jarrard et al., 2027). The target thermal inactivation approach is expected to prevent overheating while still being effective toward PL and PA inactivation.

In conclusion, the WPI substitution in fresh milk protein dispersions led to reductions in apparent PL activities, but not PA activities. These observations likely resulted from different casein- and whey-induced interference effects on the enzymatic assays used. In heat-treated samples, neither WPI substitution nor WPH substitution caused distinctive PL and PA inactivation behavior as compared with the MPC control, likely due to the fortified whey protein content in the control sample already induced near saturated inhibition effects on PL and PA. Proteolysis characterization in general followed enzyme activity results. However, the retention of intact casein in fresh samples with WPI substitutions was less than would be expected based on the enzymatic assay results, in which WPI substitution resulted in lower apparent PL activities, and thus greater retention of caseins would be expected. This can be either explained by whey protein interference on the PL enzymatic assay or a promotional effect on PA activity from WPI. The WPH substitution did not result in any improvements on proteolysis stability after the extreme storage condition for both fresh and heat-treated samples in comparison to the control. Compared with manipulations of whey protein proportion and composition, a proper heat treatment would be a more feasible and effective approach of improving proteolysis stability of high-protein model beverages primarily made from MPC. An optimal “preheat” treatment of a UHT process for acceptable proteolysis stability of RTD high-protein beverage still needs to be investigated.
